# Proceedings of the Fourth Biennial Conference of the Society for Implementation Research Collaboration (SIRC) 2017: implementation mechanisms: what makes implementation work and why? part 1

**DOI:** 10.1186/s13012-018-0714-0

**Published:** 2018-03-20

**Authors:** Cara C. Lewis, Cameo Stanick, Aaron Lyon, Doyanne Darnell, Jill Locke, Ajeng Puspitasari, Brigid R. Marriott, Caitlin N. Dorsey, Madeline Larson, Carrie Jackson, Jordan Thayer, Callie Walsh Bailey, Rebecca Lengnick-Hall, Shannon Dorsey, Sara J. Landes

**Affiliations:** 1 Kaiser Permanente Washington Health Research Institute, Seattle, WA USA; 2 Department of Psychological and Brain Sciences, Indiana University, Bloomington, IN USA; 3 Department of Psychiatry and Behavioral Sciences, University of Washington, Seattle, WA USA; 4 Hathaway-Sycamores Child and Family Services, Pasadena, CA USA; 5 UW Autism Center, University of Washington, Seattle, WA USA; 6 Department of Psychiatry and Psychology, Mayo Clinic, Rochester, MN USA; 7 Department of Psychological Sciences, University of Missouri, Columbia, MO USA; 8 Department of Educational Psychology, University of Minnesota, Minneapolis, MN USA; 9 Department of Psychology, West Virginia University, Morgantown, WV USA; 10 Suzanne Dworak-Peck School of Social Work, University of Southern California, Los Angeles, CA USA; 11 Department of Global Health, University of Washington, Seattle, WA USA; 12 Department of Psychiatry, College of Medicine, University of Arkansas for Medical Sciences, Little Rock, AR USA; 13 Central Arkansas Veterans Healthcare System, Little Rock, AR USA

## Introduction

The Society for Implementation Research Collaboration (SIRC) got its start in 2010 as the “Seattle Implementation Research Conference” National Institute of Mental Health (NIMH)-funded conference series. SIRC is now a self-funded society that hosts one of the top 15 most attended conferences in dissemination and implementation science [1]. SIRC’s goal is to foster communication and collaboration among implementation researchers, research teams, implementation practitioners, and other community stakeholders. This supplement summarizes the conference and includes published abstracts from the fourth biennial conference held in Seattle, Washington, USA on September 7–9, 2017.^1^ This conference brought together nearly 400 attendees from across the globe with diverse interests in research, policy, and practice to share learnings from their innovative work.

The 2017 conference sought to address a question central to the success of implementation efforts: “What makes implementation work and why?” There is evidence to suggest that implementation strategies tailored to the context in which they are enacted may be more effective than standardized approaches, but it is unclear how to best tailor [2–4]. As a result of this uncertainty, strategies are often mismatched to determinants of practice (e.g., training, an intrapersonal-level strategy, is inappropriately utilized to target poor culture, an organizational-level determinant [5]), and thus fail to elicit the desired outcome. Without understanding *how* implementation strategies work, the generation and use of increasingly complex and costly implementation efforts will likely fail to yield enhanced impact [6]. This conference sought to call the field to action toward mechanisms-focused research. By better understanding mechanisms of action (i.e., the process or event through which an implementation strategy operates to affect desired implementation outcomes [7]), the field will be poised to build effective, robust, and pragmatic implementation strategies that optimize outcomes. SIRC believes the field is ready to address this challenge; the presentations in this supplement highlight advances in this research direction.

## Preconference invited implementation development workshops

SIRC hosted three implementation development workshops (IDWs) for 53 invited members of the SIRC Network of Expertise. IDWs are designed for presenters to get feedback on implementation projects that are in development. Approximately a dozen attendees, multiple presenters, one facilitator, and two note takers filled each workshop room. Consistent with our published methodology [8], each presenter was offered 45 min of which they were encouraged to spend 10–20 min presenting their project/proposal. The presenter provided the group with a 1-page description of their project and three questions to guide the discussion. No technology was allowed. A facilitator-managed time and coordinated discussion to maximize the number of unique points of feedback. Note takers recorded all of the feedback, which was later provided to presenters along with any written feedback from attendees.

Based on feedback from 22 (42%) of the participants, all agreed that they learned new information and could apply this new knowledge to their own work. Additionally, the majority of respondents (*N* = 19; 86.36%) believed they left the IDW having a firmer grasp of the principles and methods of implementation research. Furthermore, a unique feature of the IDW is the limited use of technology, a feature that 17 (77.27%) respondents agreed was helpful in getting to the issues, with the remaining five participants expressing no opinion on the use of technology. When asked what was most helpful, expert input was a commonly expressed theme, which is exemplified in this quote by a presenter, “The critical feedback from D&I experts was absolutely invaluable. It is a rare and wonderful opportunity to have so many great minds constructively critiquing your work. My proposal will be much stronger as the result of the session.”

## Preconference open workshops

After the IDWs, SIRC hosted four preconference workshop sessions available to all conference attendees. The workshops aligned with SIRC’s goals of advancing research-practice partnerships and improving implementation science methodology. Two of the workshops highlighted novel research design and methodology, along with suggestions for research and practice applications: (1) *An Overview of Old and New Design and Analysis Methods for Causal Inference in Implementation Research* (presented by Donna Spiegelman), and (2) *Using Concept Mapping in Implementation Science and Practice: Methods, Applications, and Opportunities* (presented by Byron Powell and Greg Aarons)*.* SIRC offered a third workshop, paneled by a diverse group of practice leaders, policy-makers, and practitioners, focused on *Capacity Building to Sustain Implementation of EBPs: Perspectives from the Macro, Meso, and Micro Levels* (presented by Nancy McDonald, Helen Best, Ron Gengler, Dan Fox, Matthew Ditty, and Maria Monroe-DeVita)*.* These workshops allowed attendees to “dig deep” into contemporary implementation research and practice topics with leaders in the field.

Consistent with SIRC’s goal of advancing student involvement in implementation science, a fourth workshop, involving student and faculty presenters, provided information about obtaining student funding for dissemination and implementation research: *Finding and Securing D & I Research Funding for Students and Postdocs* (panelists included Shannon Dorsey, Bryce D. McLeod, Christopher Kemp, Kayne Mettert, Elena Navarro, and Miriam Rafferty)*.* Presenters focused on F31, F32, and diversity supplement funding mechanisms offered through the National Institutes of Health. Four students who successfully secured D&I research funding and two faculty members who served as student mentors and grant reviewers for these mechanisms shared their experiences. The workshop began with a brief overview of D&I funding mechanisms for undergraduate students, graduate students, post-bacs, and post-docs. Next, panelists described their personal experiences and provided practical information about the proposal preparation and selection processes. The workshop concluded with an open questions-and-answer session.

## Main conference summary

The main conference began with two plenary presentations. The first plenary was co-led by David Chambers, Nate Williams, and Cara Lewis in which they reviewed the historical context of the field, offered terms and definitions, and discussed results from two mechanism-focused systematic reviews [9] (Lewis, Boyd, Walsh-Bailey, et al.: A systematic review of empirical studies examining mechanisms of dissemination and implementation in health, in preparation). The second plenary was a series of six IGNITE presentations, which are 5-min talks consisting of 20 slides each that auto-advance every 15 s. This series of IGNITEs highlighted global implementation efforts and community-partnered research in various service settings (e.g., intermediary organizations, schools). The 2017 meeting also featured three additional plenaries book-ending each day: (1) using facilitation to implement clinical innovations (JoAnn Kirchner); (2) mechanisms of behavior change techniques (Marie Johnston); and (3) a symposium on methods for tailoring implementation strategies in behavioral health.^2^ In addition, the conference included six breakout sessions, each with three to four presentations from a variety of speakers including practice leaders, policy-makers, and researchers (students, new, and established investigators). The breakout sessions addressed multiple content areas including (1) individual-level determinants and strategies; (2) digital tools to support the implementation of effective practices; (3) connecting research to policy for enhanced implementation; (4) tailoring evaluation through innovative methodologies such as social network analysis and mixed methods; (5) organizational determinants in implementation. One breakout featured the inaugural poster teasers session that offered previews of a subset of the poster presentations.

## Committee membership and abstract review procedures

The Program Committee worked to ensure that the abstract review criteria were fair, transparent, and prioritized translational efforts.^3^ The criteria consisted of (1) Background (i.e., how succinctly and convincingly the authors outlined the rationale for the proposed study, including the problem the project set out to address); (2) Methodology and Research Design (i.e., for research-based submissions, the extent to which methodology fits the scientific question; methods and analysis are clearly stated; strong fit between aims, design, and analysis; well-executed; minimal limitations; for practice-based submissions, could include descriptions of context in place of research design); (3) Results and Conclusions (i.e., extent to which results were clearly stated, relevant statistics were reported, and the conclusion addressed the significance or implications of the results); (4) Implementation Focus (i.e., the degree to which the abstract reflects work that was squarely on the topic of implementation); and (5) Presentation Fit with Conference Theme, “Implementation Mechanisms: What Works and Why?” Each abstract was subjected to a double blind review.^4^

The SIRC 2017 conference received 195 individual submissions, which was more than double the number of submissions received for each of the previous three conferences. The Program Committee attempted to prioritize the following: maximizing the number of acceptances as in years past, reducing the number of competing presentations and number of possible break-out sessions, maintaining high quality presentations, and balancing researcher-generated with practice/provider-generated presentations. Ultimately, 60 presentations were accepted, either as plenaries or breakout symposia. An additional 60 presentations were accepted as posters, and 15 of those were also elevated into a breakout session as poster ‘teasers.’ The poster teaser format allowed presenters to orally present highlights from their work utilizing three PowerPoint slides, and conference participants were encouraged to further follow up with authors during the formal poster session reception.

SIRC is sensitive to the fact that an ironic gap may be emerging wherein our best methods are not translating from science into practice. To advance efforts to bridge research and practice in implementation science, our practitioner/policy/practice leader task force developed a new award, the Translational Award, for SIRC 2017 to encourage researchers to articulate the practice implications of their work. Specifically, we asked our presenters to incorporate at least 1–2 slides in their presentation that offered practice and/or policy implications of the study findings and, given that D&I research is often heavily context- and evidence-based practice (EBP)-dependent, we asked presenters to consider how findings may generalize to other contexts and other EBPs. This year’s winning presentation was entitled *Coordinated Knowledge Systems: Enhancing the Use of Evidence in Clinical Decision Making*, by Kimberly Becker, Alayna Park, and Bruce Chorpita. The awards committee hopes to expand on applauding the translational work of presenters at SIRC 2019, perhaps offering multiple awards for both empirical and practice-oriented presentations that excel at articulating applied and generalizable knowledge gained from the work.

In addition to the inaugural Translational Award, two standing awards were presented at SIRC. One award was student-focused, designed to recognize research led by a student at either the undergraduate, post-baccalaureate, or graduate level. Prerna Martin received the Student Award for her poster *Evaluating the Impact of a Tailored Middle-Manager-Level Pilot Facilitation Intervention to Improve Implementation of Evidence-Based Practices in Community Mental Health*. The second was new investigator-focused, designed to recognize research led by an investigator within 10 years of PhD receipt. Joanna Moulin received the New Investigator Award for her presentation *Development and Testing of a Brief EBP Implementation Intentions Scale Using Rasch Analysis.*

## Practitioner groups

Since its inception, a critical means of realizing SIRC’s mission has been to incorporate practitioners in conference and between-conference activities. Initially, we convened a task force to champion the interests of a variety of practitioner-type roles to build the practitioner membership, shape the conference, and inform initiatives. Based on feedback from practitioners, for the first time at SIRC 2017, we invited practitioners to a boxed lunch with one of three practice groups: policy-makers, intermediaries, and providers, to explore whether SIRC could better engage and serve practice partners by bringing together folks who do similar work, often by the nature of their roles. There was great enthusiasm for these practice groups, which have since formalized and are co-led by a researcher and practitioner. These groups pursue independent initiatives and will come together around conference planning, working closely with the new SIRC officer, the Practitioner Program Chair, to insure relevance of the conference to research-practice partnerships and our practitioner members.

## Networks of Expertise (NoE)

The New Investigator Network of Expertise (NoE) consists of junior investigators or researchers new to implementation science. New Investigator NoE members can be involved in several SIRC initiatives, which include presenting implementation works-in-progress and grant proposals at the IDW and participating in the mentorship program that aims to connect junior or new investigators with established investigators in implementation science. There are currently 74 members within the New Investigator NoE. In the past 2 years, there were 14 mentor matches made where each pair decides on the focus of their mentorship program, which might be on grant writing, building an implementation research agenda, professional development, and other relevant issues that will advance their career within implementation science.

The Student Investigator NoE consists of undergraduates, post-baccalaureates, and graduate students learning implementation research. There are currently 31 members in the Student Investigator NoE. This NoE is committed to facilitating the advancement of students who aim to focus their work on implementation science. Student investigator initiatives include showcasing student investigator work at SIRC conferences, offering student-focused conference events and activities, opportunities to participate in IDWs, and a mentoring program. In the mentorship program, student investigators are paired with and mentored by a more advanced implementation scientist in the New Investigator NoE, with 13 mentor matches made so far.

## Journal update

As a component of the plenary session devoted to updates on SIRC initiatives, an update was provided on the developing SIRC journal, tentatively titled *Behavioral Health Implementation Research (BHIR)*. BHIR is being developed to (a) address an identified need in the field for a publication that is dedicated to settings, outcomes, or policies/practices specific to behavioral health implementation, and (b) to reduce the budding science-to-practice gap in implementation. The BHIR update detailed a full overview of the journal development activities conducted by the steering committee since the last SIRC conference. Activities included a survey of implementation research and practice stakeholders (*N* = 109), development of a comprehensive journal concept paper, discussions with potential publishers, and the formation of an international planning committee (composed of approximately 25 implementation researchers and practitioners). The planning committee met at the SIRC 2017 conference, during which they engaged in structured discussions about scope and structure, voting in support of establishing BHIR as an independent journal, and confirming its behavioral health orientation. The BHIR planning committee is now meeting regularly to advance the journal by developing a publisher agreement, by establishing an initial editorial board, and by finalizing the timeline for launch.

## Summary

This supplement offers a compilation of the abstracts of the oral and poster presentations from the 2017 Society for Implementation Research Collaboration (SIRC) Conference, “Opening Pandora’s Box-Implementation Mechanisms: What Makes Implementation Work and Why?” The dissemination of conference products serves to enhance cross-sectoral learning in the field of implementation science and to advance rigorous and pragmatic research. Furthermore, SIRC aims to expand the accessibility of conference materials to those not in attendance by making them available in an open access publication, as well as on the SIRC website^1^.

## Abbreviations

BHIR: Behavioral Health Implementation Research
EBP: Evidence-based practice
IDW: Implementation development workshop
NoE: Network of Expertise
SIRC: Society for Implementation Research Collaboration

## Footnotes

1 The presentation slides are available on the SIRC website: https://societyforimplementationresearchcollaboration.org/4th-biennial-sirc-conference-schedule/.

2 The final symposium presentation was chaired by Byron Powell, with Laura Damschroder, Rinad Beidas, Cara C. Lewis, and Byron Powell presenting, and Amy Kilbourne serving as discussant.

3 The Program Committee consisted of the following members: Cameo Stanick, Cara C. Lewis, Shannon Dorsey, Maria Monroe-DeVita, Bianca Albers, Eva Woodward, Brad Steinfeld, Rosemary Meza, Cole Hooley, Swap Mushiana, and Madeline Larson.

4 We are grateful to have engaged the following individuals in double blind review: Rinad Beidas, Sarah Birken, Jacquie Brown, Eric Bruns, Clayton Cook, Geoff Curran, Laura Damschroder, Teresa Damush, Doyanne Darnell, Rani Elwy, Tracy Finch, Robert Franks, Joe Glass, Laurie Grealish, Kevin Hallgren, Alison Hamilton, Carolyn Heckman, Sarah Kaye, Suzanne Kerns, Melissa Kimber, Sara Landes, John Landsverk, Fabiana Lorencatto, Aaron Lyon, Nancy McDonald, Laura Murray, Natasha Pearce, Byron J. Powell, Enola Proctor, Lisa Ruble, Lisa Saldana, Sonja Schoenwald, J.D. Smith, Shannon Wiltsey Stirman, Sean Wright.
